# Integrated pan-cancer gene expression and drug sensitivity analysis reveals *SLFN11* mRNA as a solid tumor biomarker predictive of sensitivity to DNA-damaging chemotherapy

**DOI:** 10.1371/journal.pone.0224267

**Published:** 2019-11-04

**Authors:** Kevin Shee, Jason D. Wells, Amanda Jiang, Todd W. Miller

**Affiliations:** 1 Department of Molecular & Systems Biology, Geisel School of Medicine at Dartmouth, Lebanon, New Hampshire, United States of America; 2 Comprehensive Breast Program, Dartmouth-Hitchcock Norris Cotton Cancer Center, Lebanon, New Hampshire, United States of America; Peter MacCallum Cancer Institute, AUSTRALIA

## Abstract

**Background:**

Precision oncology seeks to integrate multiple layers of data from a patient’s cancer to effectively tailor therapy. Conventional chemotherapies are sometimes effective but accompanied by adverse events, warranting the identification of a biomarker of chemosensitivity.

**Objective:**

Identify an mRNA biomarker that predicts chemosensitivity across solid tumor subtypes.

**Methods:**

We performed a pan-solid tumor analysis integrating gene expression and drug sensitivity profiles from 3 cancer cell line datasets to identify transcripts correlated with sensitivity to a panel of chemotherapeutics. We then tested the ability of an mRNA biomarker to predictive clinical outcomes in cohorts of patients with breast, lung, or ovarian cancer.

**Results:**

Expression levels of several mRNA transcripts were significantly correlated with sensitivity or resistance chemotherapeutics in cancer cell line datasets. The only mRNA transcript significantly correlated with sensitization to multiple classes of DNA-damaging chemotherapeutics in all 3 cell line datasets was encoded by *Schlafen Family Member 11* (*SLFN11*). Analyses of multiple breast, lung, and ovarian cancer patient cohorts treated with chemotherapy confirmed *SLFN11* mRNA expression as a predictive biomarker of longer overall survival and improved tumor response.

**Conclusions:**

Tumor *SLFN11* mRNA expression is a biomarker of sensitivity to an array of DNA-damaging chemotherapeutics across solid tumor subtypes.

## Introduction

In the emerging era of precision oncology, the molecular features of a tumor are being used to guide treatment decisions for each individual patient. “Targeted therapies” have been developed over the past 20 years that are selective for overexpressed or mutant oncoproteins in cancer cells, such as BRAF inhibitors (*e*.*g*., dabrafenib) for the treatment of patients with BRAF^V600^-mutant melanoma [1]. While the development of such tumor-targeted therapies is conceptually straightforward (*i*.*e*., drug targets aberrant protein), approaches to leverage molecular features of tumors to refine the use of “non-targeted therapies” (*i*.*e*., conventional chemotherapies) remain underdeveloped. This is especially important in the current clinical environment where chemotherapy, which we broadly define herein as a small molecule that is not targeted to an oncoprotein or prescribed due to a specific genetic aberration or cancer cell lineage characteristic, remains the standard-of-care treatment for most cancer subtypes. However, there is a large heterogeneity of response to chemotherapy, and retrospective analysis of clinical response data shows that a large proportion of patients do not derive benefit from chemotherapy [2, 3]. It would thus be extremely valuable for the clinical cancer community to have biomarkers predictive of response to chemotherapeutics that could be used to risk-stratify patients and inform ideal drug choice.

Based upon the notion that cancer cell sensitivity to a chemotherapeutic may be associated with cancer cell-intrinsic molecular features, we hypothesized that molecular features of cancer cell line models could be used to identify a molecular predictor of response to chemotherapy in human tumors. Furthermore, we sought to analyze these data both in aggregate, and by chemotherapy class. We leveraged the wealth of publicly available cancer cell line gene expression and drug sensitivity data to identify transcripts that predict chemosensitivity and chemoresistance. We report the identification of Schlafen Family Member 11 (*SLFN11*) mRNA level as a biomarker predictive of response to chemotherapeutics, including topoisomerase inhibitors, alkylating agents, anti-metabolites, and anti-tumor anti-biotics in solid tumor lineages. We further show that *SLFN11* mRNA level is a tumor biomarker predictive of overall survival (OS) and enhanced tumor response in breast, lung, and ovarian cancer patients treated with these chemotherapeutics.

## Materials and methods

### Data acquisition

Robust Multi-array Average (RMA)-normalized transcriptomic data and drug sensitivity data [area under the curve (AUC) and IC_50_] were downloaded for A) 860 cancer cell lines and 481 drugs from the Cancer Therapeutics Response Portal (CTRP) [4], B) 1,065 cancer cell lines and 266 drugs from the Genomics of Drug Sensitivity in Cancer (GDSC) database [5], and C) 60 cancer cell lines and 783,538 compounds and drugs from the National Cancer Institute 60 (NCI60) database [6]. Analyses were limited to cell lines from solid tumor lineages (S1 Fig). In addition to *SLFN11* mRNA expression in cell lines, tumor *SLFN11* expression RNA-sequencing data generated by the TCGA Research Network (http://cancergenome.nih.gov/) [7–9] were downloaded from the National Cancer Institute Genomic Data Commons Data Portal (https://portal.gdc.cancer.gov/; Data Type “Gene Expression Quantification”) and analyzed. Venn diagrams showing overlap between cancer cell lines and genes in the databases were created using the Venn Diagram Plotter (https://omics.pnl.gov/software/venn-diagram-plotter).

### Correlation analyses

Pearson’s correlations between expression of all individual mRNAs and drug sensitivity (AUC or IC_50_) were performed for all solid tumor cell lines in response to treatment with each alkylating agent, anti-metabolite, anti-tumor anti-biotic, microtubule inhibitor, and topoisomerase inhibitor available in the CTRP, GDSC, and NCI60 databases (Table 1). To determine mRNAs that were associated with sensitivity or resistance across drug classes and between databases, we used the following criteria to select genes: Pearson correlation of R>0.2 in >50% of drugs in the chemotherapeutic class in ≥2 of the 3 databases. Pearson’s correlations were also used to compare *SLFN11* mRNA expression between overlapping cell lines in each of the databases. Statistical analyses were performed using Graphpad Prism.

**Table 1:** Chemotherapeutics in the 3 cancer cell line datasets.

Alkylating agents	CTRP	GDSC	NCI60
**Bendamustine**	+		+
**Carboplatin**	+		+
**Carmustine**			+
**Chlorambucil**	+		+
**Cisplatin**		+	+
**Cyclophosphamide**	+		+
**Dacarbazine**	+		+
**Estramustine**			+
**Ifosfamide**			+
**Lomustine**			+
**Melphalan**			+
**Nitrogen mustard**			+
**Oxaliplatin**	+		+
**Pipobroman**			+
**Procarbazine**	+		+
**Streptozocin**			+
**Temozolomide**	+	+	
**Thiotepa**			+
**Triethylenemelamine**			+
**Uramustine**			+
**Cytoskeleton inhibitor**			
**Cytochalasin**	+		
**Docetaxel**	+	+	+
**Epothilone B**		+	
**Itraconazole**	+		
**Ixabepilone**			+
**Paclitaxel**		+	+
**Parbendazole**	+		
**Vinblastine**		+	+
**Vincristine**	+		+
**Vinorelbine**		+	+
**Anti-metabolites**			
**5FU**	+		
**6-Mercaptopurine**			+
**Cladribine**			+
**Clofarabine**	+		+
**Cytarabine**	+	+	+
**Decitabine**	+		
**Floxuridine**			+
**Fludarabine**			+
**Fluorouracil**			+
**Gemcitabine**	+	+	+
**Methotrexate**	+	+	
**Anti-tumor antibiotics**			
**Actinomycin D**			+
**Bleomycin**	+	+	
**Daunorubicin**			+
**Doxorubicin**		+	+
**Epirubicin**			+
**Idarubicin**			+
**Mitomycin**	+	+	+
**Mitoxantrone**			+
**Valrubicin**			+
**Topoisomerase inhibitor**			
**Camptothecin**		+	
**Etoposide**	+	+	+
**Irinotecan**			+
**SN-38**	+	+	+
**Teniposide**	+		+
**Topotecan**	+		+

### Clinical data analyses

The NCBI Gene Expression Omnibus was queried for datasets that included A) treatment-naïve primary solid tumor gene expression profiles, B) treatment of patients with cytotoxic chemotherapies, and C) clinical follow-up data. The following datasets were obtained and analyzed: GSE37751 [10], GSE29013 [11], GSE37745 [12], GSE17260 [13], GSE32646 [14], and GSE63885 [15]. Of note, many datasets identified used a limited gene expression microarray platform that did not contain probes for *SLFN11*, and were thus excluded from our analyses (*e*.*g*., GSE20194, GSE20271, GSE22093, GSE23988, GSE25066, GSE41998, E-GEOD-31245). Patients were stratified by tumor *SLFN11* mRNA levels above or below the median, and survival analyses were performed using log-rank (Mantel-Cox) test. For breast cancer patients with pathologic response data, patients were stratified by pathologic Complete Response (pCR) vs. non-pCR, and tumor *SLFN11* mRNA levels were compared by *t*-test. For ovarian cancer patients with clinical response data, patients were stratified by Complete Remission (CR) vs non-CR, and analyzed as above.

## Results

### Cancer cell line database analyses reveal SLFN11 mRNA expression as a predictive biomarker of chemosensitivity

We first sought to identify transcripts with expression levels that predict response to chemotherapeutics across solid tumor subtypes. Gene expression and drug sensitivity profiles were utilized from 3 cancer cell line databases: Cancer Therapeutics Response Portal (CTRP), Genomics of Drug Sensitivity in Cancer (GDSC), and National Cancer Institute 60 (NCI60). Pearson correlation coefficients across cell lines were determined for all 2020930 individual gene-drug combinations using the 27, 17, and 47 chemotherapeutics tested in each dataset, and the 18988, 17737, and 25675 transcripts analyzed in each dataset (S1 Table). Overlap between transcripts and cell lines between the 3 datasets is shown in S1A and S1B Fig. Genes largely overlapped between datasets, with 13458 genes shared between all 3 datasets. Solid tumor cell lines had only partial overlap; among the 854, 801, and 58 solid tumor cell lines in these databases, 496 cell lines were present in ≥2 databases. Overlap between drugs in the datasets is shown in Table 1.

**Fig 1:**
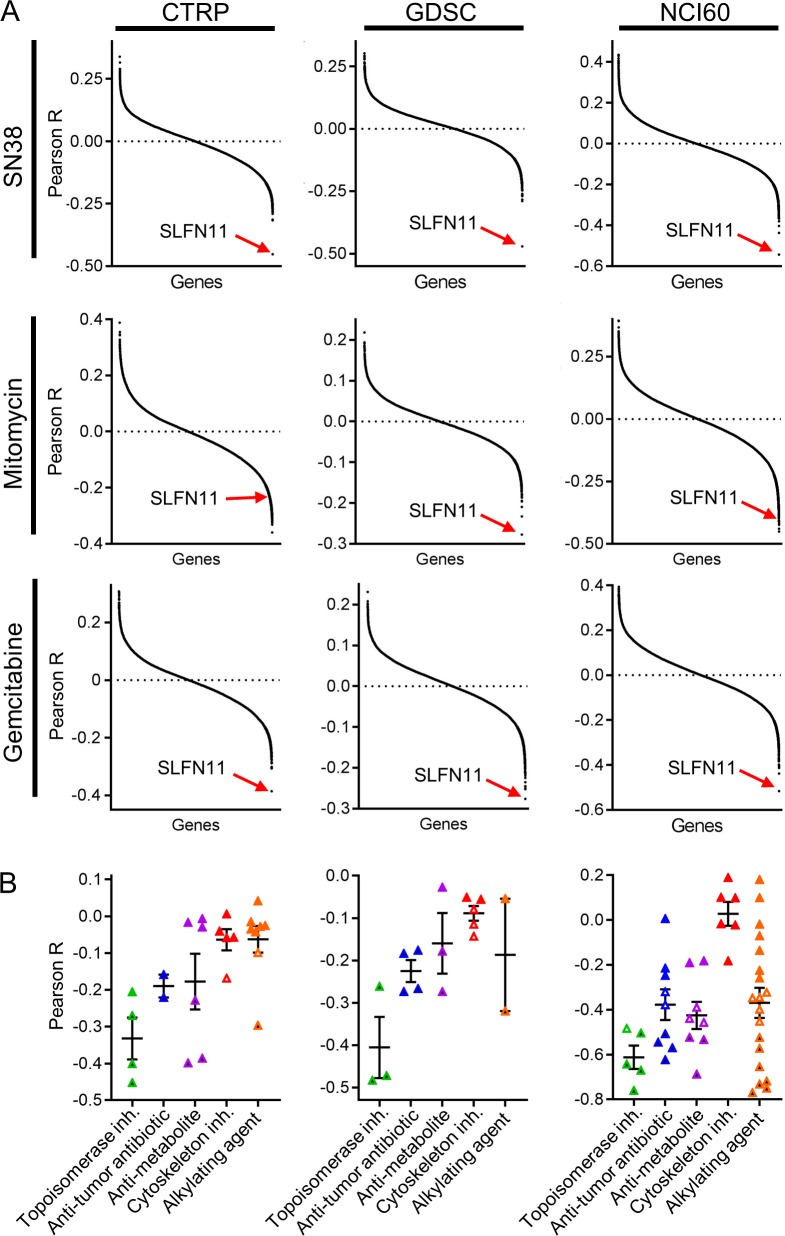
SLFN11 mRNA levels are strongly correlated with sensitivity to chemotherapeutics in cancer cells. A) mRNA levels for each gene were compared with drug sensitivity to a panel of chemotherapeutics across the CTRP (IC_50_), GDSC (AUC), and NCI60 (IC_50_) datasets. Pearson correlation values were plotted for each gene for SN38, mitomycin, and gemcitabine as representative chemotherapeutics. B) SLFN11 mRNA levels were compared with drug sensitivity as in (A) by Pearson correlation. Each point represents the IC_50_ of a given drug. Horizontal lines indicate mean ± SEM for each drug class. Black filled, white-filled, and color-filled symbols indicate p≤0.001, p≤0.05, and p>0.05, respectively.

**Fig 2:**
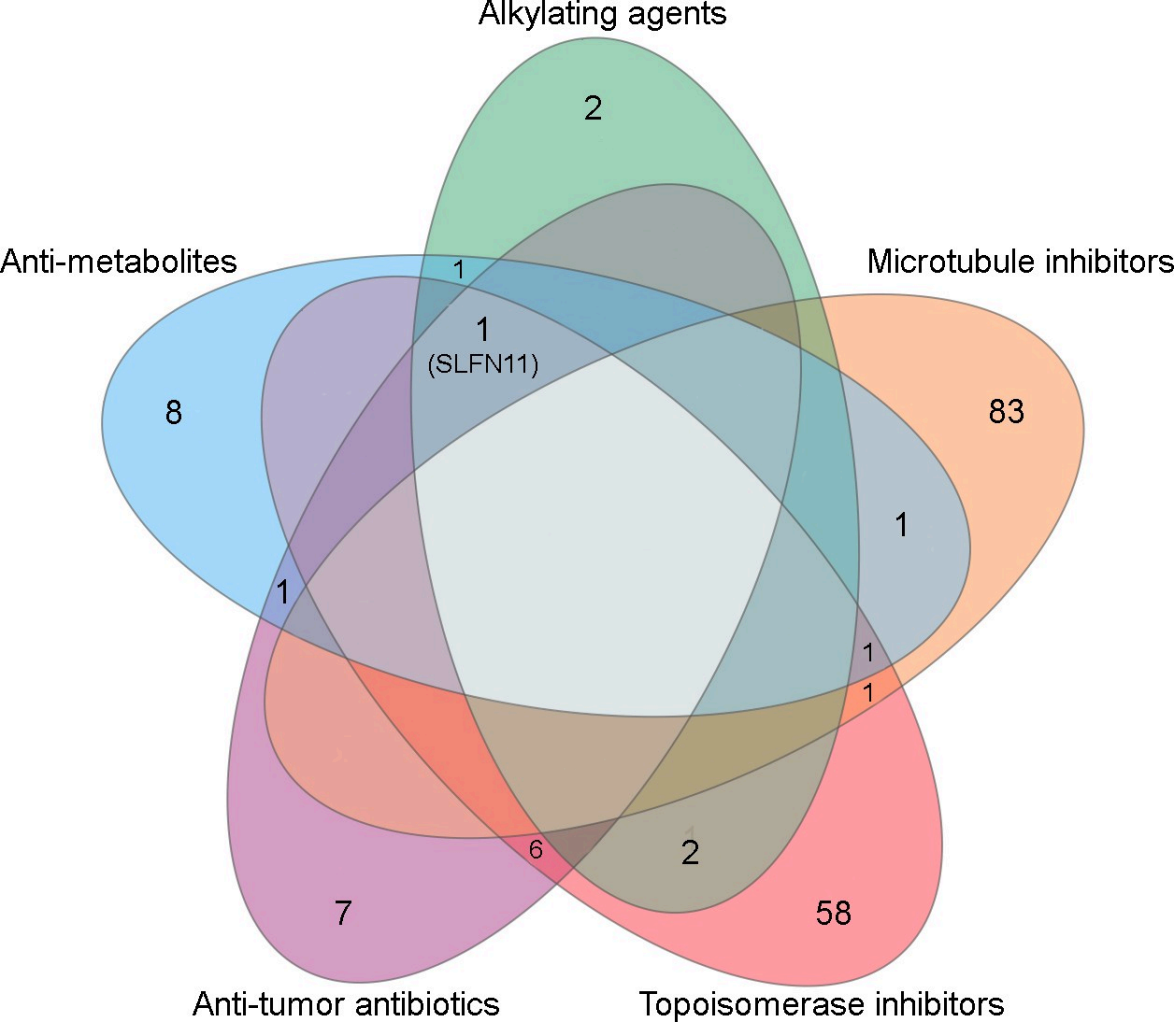
SLFN11 mRNA expression is commonly associated with chemosensitivity in cancer cell lines. Numbers of genes with expression correlated with sensitivity (R≤-0.2) or resistance (R≥0.2) to >50% of drugs within a class in ≥2 databases (CTRP, GDSC, or NCI60) are indicated. Genes are listed in Table 2.

Fig 1A shows results from transcriptome-wide correlation analyses of 3 representative drugs (SN38, mitomycin C, and gemcitabine) that were present in all 3 datasets and from 3 classes of chemotherapeutics (topoisomerase inhibitors, anti-tumor antibiotics, and anti-metabolites, respectively); each point represents one gene-drug combination. *SLFN11* ranked as the most strongly negatively correlated transcript with ln(IC_50_) or AUC values for gemcitabine and SN38 in all 3 datasets, and ranked highly for negative correlation with mitomycin C response values (all Pearson p<0.001); these findings suggest that high *SLFN11* mRNA levels are predictive of increased chemosensitivity. When considering drugs by class of chemotherapeutic, *SLFN11* was the only transcript correlated (R≤-0.2) with sensitivity to at least half of tested chemotherapeutics in 4/5 classes tested (*i*.*e*., alkylating agents, anti-metabolites, anti-tumor anti-biotics, and topoisomerase inhibitors) in ≥2 databases (Fig 2 and Table 2). Zoppoli et al. previously found that 23 transcripts including *SLFN11* are associated with sensitivity to topoisomerase inhibitors [16]; comparison with genes in Table 2 indicates that 2, 3, 2, 0, and 4 of those 23 genes are associated with sensitization to alkylating agents, anti-metabolites, anti-tumor anti-biotics, microtubule inhibitors, and topoisomerase inhibitors in our analyses, respectively. Correlation values for *SLFN11* transcript levels with each drug are shown by class of drug in Fig 1B; GDSC reported drug sensitivity data as both ln(IC_50_) and AUC (S2A Fig), which both showed that *SLFN11* transcript level is highly correlated with chemosensitization. Correlation values for individual chemotherapeutics are shown in S2B–S2E Fig. When evaluating transcripts associated with chemosensitivity or chemoresistance as gene sets, we did not observe hallmark gene sets consistently associated with sensitivity/resistance across drug classes (S3 Fig).

**Table 2:** SLFN11 mRNA expression is commonly associated with chemosensitivity in cancer cell lines. All genes listed here were correlated with sensitivity (R≤-0.2) or resistance (R≥0.2) in >50% of drugs in class in ≥2 databases (CTRP, GDSC, or NCI60). Bold indicates overlap in all 3 databases. Underline indicates overlap with topoisomerase-sensitizing genes reported in ref. [16].

Drug class	Genes associated with sensitivity	Genes associated with resistance
**Alkylating agents**	PFAS, **SLFN11**	LSR, MST1R, OSBPL2, PLEKHA7
**Anti-metabolites**	CSNK2A2, EP400, EXOSC2, LIMD2, METAP2, MRPS27, NOB1, NPM3, PFAS, PPP1CC, **SLFN11**, TRAP1, ZNF280C	PTTG1IP
**Anti-tumor Antibiotics**	ATF1, BLMH, METAP2, MEX3B, MIR600HG, NSL1, RGS16, RTN1, **SLFN11**, TATDN3, ZFP1	C3orf52, EIF6, TPD52L2, TPRG1L
**Microtubule Inhibitors**	AGPAT5, ALMS1, ANP32B, ATXN7L2, C15orf61, C2orf44, CECR5, CEP85, CNTRL, COQ3, CRLF3, DKC1, EXOSC2, FBXO45, GMEB1, GNL2, HMGXB4, HNRNPR, IKBKAP, ITGB3BP, KBTBD6, KDM1A, KIF2A, MAK16, NASP, NCBP1, NUP160, NUP188, NUP88, ODF2, OIP5, ORC1, OTUD3, POLA1, POLR1E, PWP2, RCC2, RMI1, RPA2, RUVBL1, SKP2, SNRPA, SPAST, SPATA5L1, TAF5, TBPL1, TTF1, TTI2, TUBGCP4, TXLNG, UPF3B, WDR18, WRAP73, ZNF142, ZNF184, ZNF227, ZNF280C	ABCB1, ALDH3B1, BCL2L1, BICC1, CFLAR, COMMD7, DRAM1, DYSF, EHD1, GALNT10, GLS, GRAMD3, ITGA3, LASP1, LEPROT, MGLL, MVP, NPC2, PHLDA3, PLK2, SLC35F5, SUMF1, TGM2, THBS1, TNFRSF12A, TRAM1, UGCG, UXS1, ZFP36L1
**Topoisomerase Inhibitors**	ANGEL2, ANP32B, ANTXR1, ATG4C, BCAT1, BLMH, BPTF, CAPRIN1, CASC3, CSNK1E, DLG4, DNAJC7, DSE, EP400, FAM129A, HAND2, HMX2, KHDRBS1, KIF5C, MEX3B, MRC2, MRPL42, NAP1L1, NSL1, NUCKS1, NUDT10, PHF21A, PPM1E, PSIP1, RAB39B, RTN1, SENP1, **SLFN11**, SNHG1, SNRPF, TCF4, TGFB1, TLK2, VBP1, WASF1, ZFHX4, ZFP1, ZNF280C, ZNF483	CLDN4, LIPH, SLC35A2, CEACAM5, CHKA, ELF3, EPCAM, LSR, MANSC1, MISP, OVOL2, PPFIBP2, PRR15, RAB11FIP4, RAB17, RASEF, RDH13, SHROOM3, ARHGEF5, B4GALT4, FUCA1, GIPC1, OSBPL2, TMEM184B, TNFRSF21, TPRG1L

Gene expression profiles in these 3 cell line databases were generated using different platforms. To determine whether the use of different platforms affected detection of *SLFN11* mRNA, Pearson correlation coefficients were determined for *SLFN11* mRNA values among all solid tumor cell lines common between databases. *SLFN11* expression was significantly correlated (all *p*<0.001) between all pairs of datasets (S4 Fig). Gene expression was compared between cancer cell lineages across cell line databases, and for human tumors in The Cancer Genome Atlas (TCGA). *SLFN11* expression was consistently higher and lower in certain cancer types (e.g. kidney and large intestine/colon, respectively) (S5A–S5D Fig). Tang *et al*. also reported that *SLFN11* levels are highest in acute myeloid leukemia compared to various solid tumors subtypes evaluated in TCGA [17].

### SLFN11 mRNA expression is prognostic of improved patient outcome following adjuvant chemotherapy

**Fig 3:**
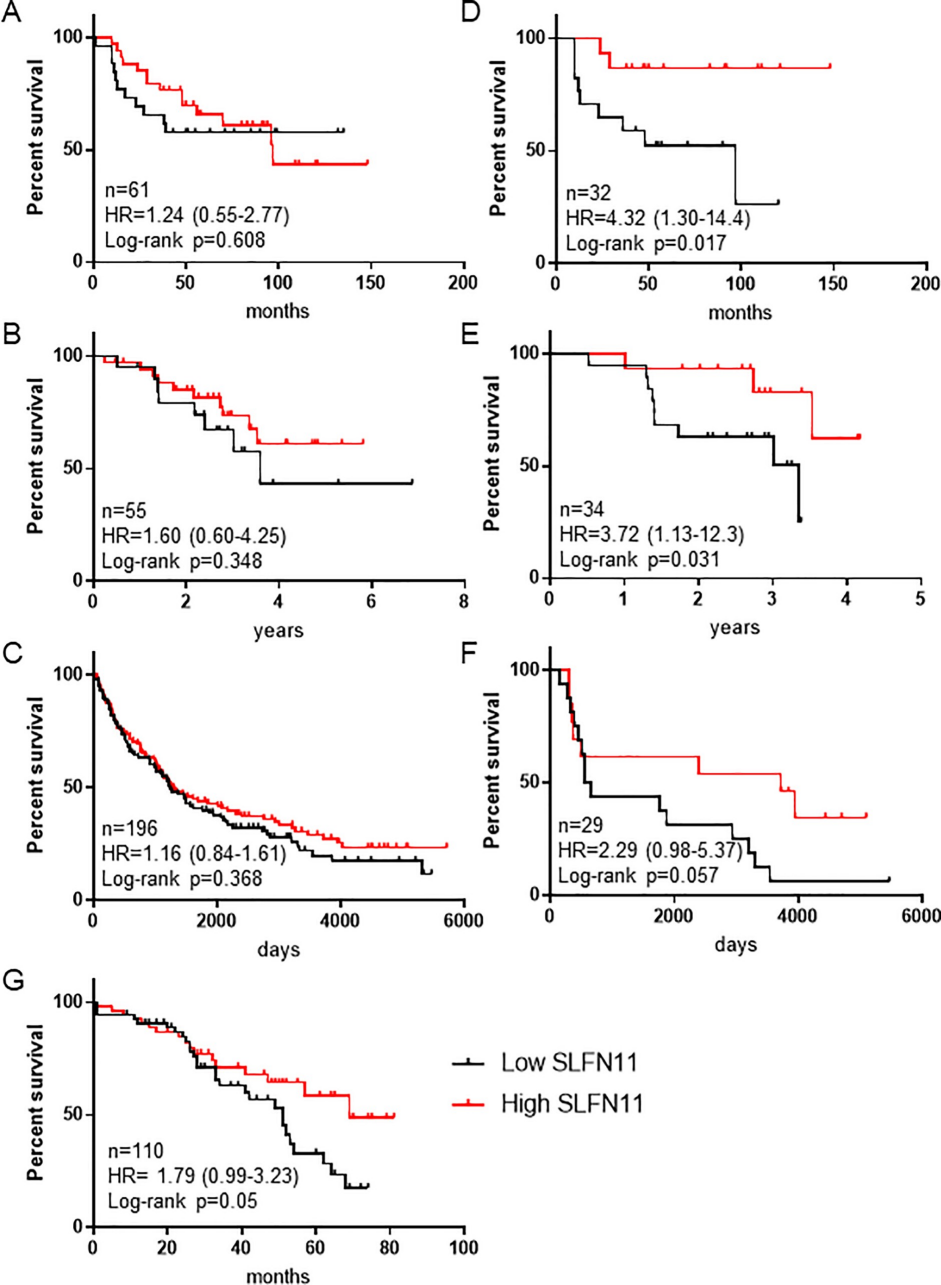
SLFN11 expression is associated with improved survival outcomes in breast, lung, and ovarian cancer patients treated with chemotherapy. RNA expression and survival data were obtained for primary breast, lung, and ovarian tumors from 4 datasets containing information from 61 breast cancer patients (A/D), 55 and196 lung cancer patients (B/E and C/F, respectively), and 110 ovarian cancer patients (G). Patients were dichotomized into High vs. Low tumor SLFN11 mRNA based on expression above or below the median. Survival analyses were performed for all patients in aggregate in (A-C), and only for patients who received chemotherapy (D-G). Groups were compared by log-rank test.

Survival analyses were performed using 4 breast, lung, and ovarian cancer clinical datasets. When available, survival analyses were performed as a total aggregate (Fig 3A–3C), and repeated after excluding patients who did not receive cytotoxic chemotherapies (Fig 3D–3G). Patients were dichotomized based on median tumor *SLFN11* mRNA expression.

In a dataset of 61 unselected breast cancer patients [10], aggregate survival analysis showed no significant difference in overall survival (OS) between patients with high vs. low tumor *SLFN11* expression (HR = 1.24, p = 0.608) (Fig 3A). In two datasets of 55 and 196 non-small-cell lung cancer patients [11, 12], there was no significant differences in OS between patients with high vs. low *SLFN11* expression (HR = 1.60 and 1.16, p = 0.348 and 0.368, respectively) (Fig 3B and 3C). Similarly, *SLFN11* expression was not prognostic of OS in 18/21 cancer subtypes included in TCGA (S6 Fig). When analyzing only breast cancer patients who received chemotherapy (drugs not specified), there was a significant OS benefit in patients with high *SLFN11* expression (HR = 4.32, p = 0.017) (Fig 3D). Similarly, when analyzing only lung cancer patients who received adjuvant chemotherapy (drugs not specified), there was a significant OS benefit in patients with high *SLFN11* expression in one dataset (HR = 3.72, p = 0.031, Fig 3E) and a trend towards benefit in a second dataset (HR = 2.29, p = 0.057, Fig 3F). Finally, in an ovarian cancer dataset of 110 patients, all of whom received cisplatin-based chemotherapy, high *SLFN11* expression was associated with longer OS (HR = 1.79, p = 0.05) (Fig 3G).

### Patients with tumors highly responsive to chemotherapy have high SLFN11 transcript levels

**Fig 4:**
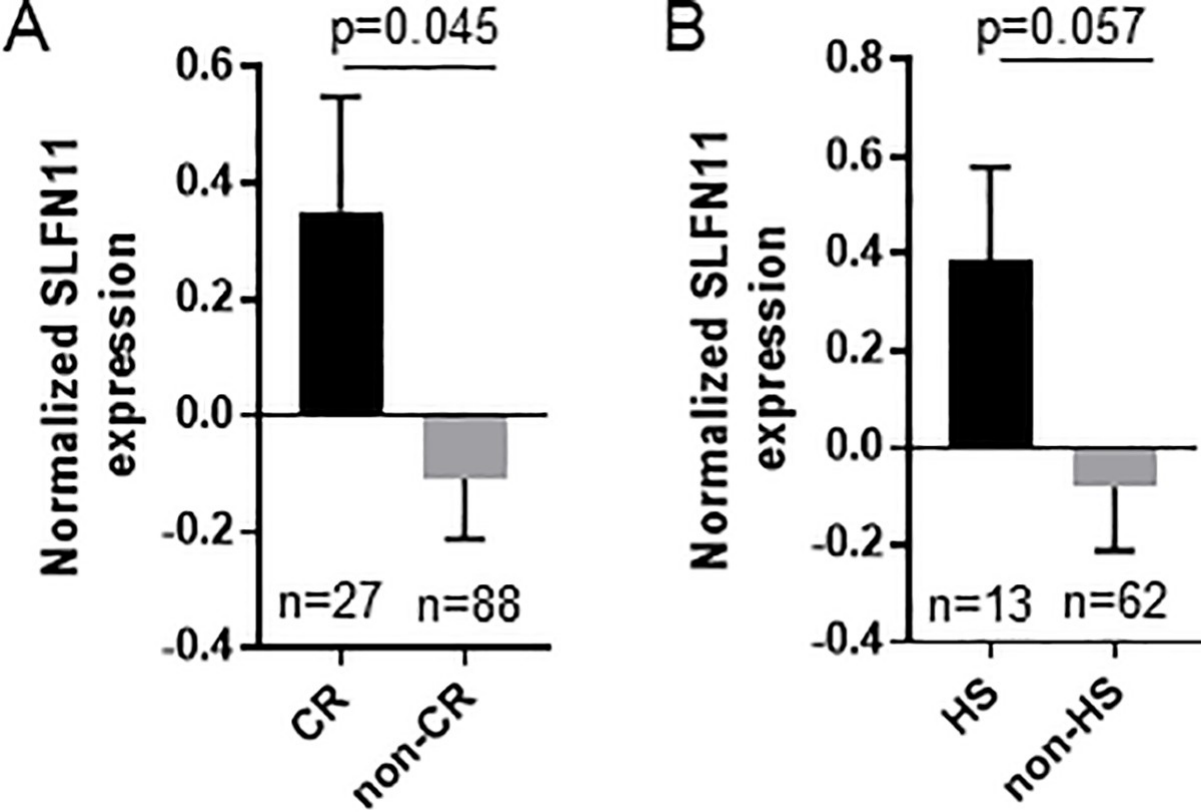
Patients with tumors highly responsive to chemotherapy have high SLFN11 transcript levels. Z-score normalized RNA expression and tumor response data were obtained for primary breast and ovarian tumors from 2 datasets containing information from 115 breast cancer patients (A) and 75 ovarian cancer patients (B) treated with neoadjuvant chemotherapy. Breast cancer patients were divided into patients who had a pathologic Complete Response after chemotherapy (pCR), to those that did not (non-pCR), and SLFN11 expression was compared by Student *t*-test with Welch’s correction. Ovarian cancer patients were divided into patients who were highly sensitive (HS; defined as DFS>732 days according to [15]) or not (non-HS), and analyzed as above.

A cohort of 115 patients with breast cancer (tumor size T1-4b; node-positive or -negative; stage IIA-IIIB) were treated with neoadjuvant therapy consisting of paclitaxel followed by a combination of 5-FU, epirubicin, and cyclophosphamide. Following surgical removal of the primary breast tumor and lymph nodes, pathologic response to neoadjuvant chemotherapy was evaluated by histological examination of the surgical specimens. Pathologic Complete Response (pCR) was defined as no evidence of residual invasive cancer in both the breast and axilla [14]. Comparison of *SLFN11* transcript levels in baseline (pre-treatment) tumor biopsy specimens showed that patients with CR had significantly higher *SLFN11* expression than those that did not (p = 0.045) (Fig 4A). We then analyzed data from a cohort of 75 patients with ovarian cancer treated with a platinum-containing regimen (carboplatin or cisplatin with cyclophosphamide or taxane) and monitored for response to chemotherapy [15]. For our analysis, these patients were divided into patients who were highly sensitive (HS) or non-HS. Comparison of *SLFN11* transcript levels in these tumors, while statistically limited by sample size, also demonstrated a trend towards higher levels of *SLFN11* expression in patients with CR vs. non-CR (p = 0.057) (Fig 4B).

## Discussion

Traditional chemotherapeutic agents were first used in the 1940s with nitrogen mustards and folic acid antagonists. Since their inception, treatment with these agents and their derivatives alone and in combination has become the standard of care for the majority of cancer subtypes. While new targeted agents exhibiting more favorable adverse event profiles have emerged as a result of precision oncology research, these agents have not replaced traditional chemotherapy. Evidence has also begun to emerge suggesting that resistance to therapy is more prevalent with tumor-targeted agents (particularly when used as single-agents) compared to DNA-damaging chemotherapeutics, which may be due in part to the ability of cancers to alter/mutate the drug target (as in the case of BCR-ABL) or bypass targeted inhibition by rewiring pathways or shifting dependence to compensatory signaling [18–21]. It is likely that the future of cancer management will include combination therapy regimens including both tumor-targeted and chemotherapeutic agents.

While chemotherapeutics are extremely effective, there remains significant heterogeneity in response between patients. We sought to identify transcripts that predict tumor response and survival outcome in patients receiving chemotherapies. Using an extensive correlation analyses involving 1190 cell lines and 56 drugs belonging to 5 different chemotherapeutic classes, we identified *SLFN11* as the transcript most strongly predictive of response to alkylating agents, anti-metabolites, anti-tumor antibiotics, and topoisomerase inhibitors, all of which induce DNA damage, but not to microtubule inhibitors. We further validated these findings in eight clinical datasets, and showed that higher levels of *SLFN11* mRNA expression in treatment-naïve primary tumors predict improved OS and tumor response to chemotherapies. These data collectively suggest that *SLFN11* mRNA has the potential to be a biomarker predictive of benefit from DNA-damaging chemotherapies, and to have a role in identifying subsets of patients who may require more or less aggressive therapeutic strategies. These findings confirm prior reports showing that high *SLFN11* mRNA or protein levels, as well as low levels of *SLFN11* promoter methylation, are predictive of improved response to DNA-damaging chemotherapies (Table 3).

**Table 3:** Prior studies that evaluated SLFN11 as a prognostic or predictive biomarker in cancer patients.

Cancer type	*n* of patients	Drugs	Conclusions	Ref.
**Ewing sarcoma**	44	Not specified	Tumors with high *SLFN11* mRNA levels were associated with longer RFS (p = 0.0046).	[43]
**Ovarian cancer**	110	Cisplatin-based chemotherapy	High SLFN11 mRNA levels were associated with better OS (p = 0.016).	[16]
**Recurrent small cell lung cancer**	104	Temozolomide + veliparib or placebo	Temozolomide + veliparib elicited longer PFS (5.7 v 3.6 months; p = 0.009) and OS (12.2 v 7.5 months; p = 0.014) in patients with SLFN11+ tumors vs. SLFN11- tumors.	[32]
**Colorectal cancer**	128	Not specified	*SLFN11* promoter methylation was prognostic of poor 5-year OS and 5-year RFS (p<0.05).	[16]
**Colorectal cancer**	237	Oxaliplatin-based chemotherapy	Among 153 patients with *KRAS*-wild-type tumors, SLFN-high tumors were associated with longer OS compared to SLFN11-low tumors (p = 0.048).	[44]
**Non-small cell lung cancer**	22	Platinum-based chemotherapy	*SLFN11* promoter hypermethylation was associated with shorter PFS (p = 0.031).	[45]
**Ovarian cancer**	41	Cisplatin or carboplatin	*SLFN11* promoter hypermethylation was associated with shorter (OS) (p = 0.006) and PFS (p = 0.003).	[45]

*SLFN11* is a member of the *Schlafen* (*Slfn*) family of genes that were originally identified as growth-regulatory genes differentially expressed during lymphocyte development [22]. To date, 6 human *Slfn* genes have been identified (*SLFN5*, *SLFN11*, *SLFN12*, *SLFN12L*, *SLFN13*, *and SLFN14*) [23]. Additionally, there are 13 splice variants of *SLFN11*, encoding different isoforms; although the gene expression platforms used herein did not distinguish between isoforms, results of our cross-platform analysis suggest that a pan-isoform measure of *SLFN11* is sufficient. Evidence suggests that SLFN11 protein has DNA/RNA helicase activity, and this protein has been implicated in inhibition of HIV-1 retroviral replication [24]. SLFN11 protein has been reported to sensitize cells to DNA-damaging agents by inhibition of homologous recombination, but these analyses had previously been restricted to analyses of single datasets or cell types [25, 26]. Thus, we speculate that *SLFN11* mRNA levels were not predictive of sensitivity to microtubule inhibitors in our study because induction of DNA damage is not the primary mechanism of anti-cancer action of these drugs [27]. To functionally implicate SLFN11 in response to DNA-damaging chemotherapeutics (topoisomerase inhibitors, an anti-metabolite, and an alkylating agent), Li *et al*. demonstrated that genetic inhibition of *SLFN11* expression caused chemoresistance [28]. Others also found that *SLFN11* knockdown conferred resistance to an alkylating agent and PARP inhibitors; these drugs induced downregulation of SLFN11 levels in cancer cells, potentially explaining the link between low SLFN11 levels and chemoresistance [29, 30]. Barretina *et al*. observed an association between *SLFN11* mRNA levels and sensitivity to topoisomerase inhibitors, but *SLFN11* knockdown did not alter chemosensitivity [31]. These disparate findings on the role of SLFN11 in chemosensitivity may be attributable in part to the use of different drugs by different research teams; furthermore, the use of continuous drug exposure paradigms in cell culture may elicit different effects than those observed in patients due to pharmacokinetic properties. Recent evidence suggests that SLFN11 protein expression has clinical utility in predicting response to PARP inhibitors: a randomized Phase II study by Pietanza *et al*. showed that patients with small cell lung cancers expressing SLFN11 had improved progression-free survival (PFS) and (OS) upon treatment with the combination of a PARP inhibitor and the alkylating agent temozolomide [32]. This was the first clinical trial demonstrating clinical utility of SLFN11 as a predictive biomarker, and warrants additional prospective, randomized clinical trials to determine utility in other clinical settings. Tang *et al*. recently demonstrated that treatment with histone deacetylase (HDAC) inhibitors increase *SLFN11* expression, which may be developed as a strategy to sensitize cancer cells to chemotherapies [17].

Several studies have identified individual molecular biomarkers associated with chemotherapy response, but these studies are typically done in specific cancer subtypes in response to one drug or combination. For example, high levels of phosphoglycerate kinase-1 (PGK1) expression have been associated with shorter survival in breast cancer patients treated with paclitaxel chemotherapy [33]. Similarly, high protein tyrosine kinase 7 (PTK7) expression has been associated with improved disease-free survival in breast cancer patients receiving taxane-based chemotherapy, but with worse disease-free survival in breast cancer or acute myeloid leukemia patients receiving anthracycline chemotherapy [34, 35]. Another study has linked high levels of DNA-dependent kinase catalytic subunit D (PRKDC) to chemoresistance in breast cancer patients treated with adjuvant chemotherapy [36]. Additional approaches have used multi-gene expression profile-based approaches to identify patients likely to respond to chemotherapy, including the Oncotype Dx 21-gene transcriptional signature in breast cancer [37–39]. While these single- and multi-gene biomarker panels may have utility in selective populations (*e*.*g*., breast cancer patients), they will likely not have the same level of external validity for the analysis of other cancer subtypes. To our knowledge, our study is the first to identify a single-gene mRNA biomarker with applicability across multiple cancer subtypes and classes of chemotherapeutics.

Importantly, this study was performed based on the notion that drug sensitivity is cancer cell-intrinsic. However, we acknowledge that there is a significant contribution to drug response mediated by the tumor microenvironment (TME). Components of the TME including stromal cells and secreted factors have been implicated in drug resistance to both chemotherapeutics and targeted agents in a multitude of cancer types [20, 21]. Similarly, TME components such as secreted factors, hypoxia and acidity have been shown to contribute to enhancing drug sensitivity in cancer [40–42]. Determining potential interplay between SLFN11 expression, the tumor microenvironment, and sensitization to chemotherapy represents a logical and clinically important next step.

In summary, we have analyzed a wealth of cell line and patient data, and uncovered *SLFN11* as a biomarker predictive of improved response and survival to multiple classes of chemotherapeutics that applies broadly to multiple solid tumor subtypes. Future investigation will involve prospective clinical trials in multiple cancer subtypes to determine whether *SLFN11* expression predicts tumor response, recurrence, progression, and survival in patients treated with topoisomerase inhibitors, alkylating agents, anti-metabolites, or anti-tumor antibiotics. Findings from this work may provide rationale for pre-screening patients prior to systemic treatment to best tailor therapy on a patient-by-patient basis.

## Supporting information

S1 Fig Genes and solid tumor cell line overlap between cell line databases. RMA-normalized basal expression profiles for cell lines was downloaded from CTRP v2 (https://ocg.cancer.gov/programs/ctd2/dataportal) and GDSC (https://www.cancerrxgene.org/downloads). Z-score-normalized gene expression profiles for cell lines was downloaded from NCI60 (https://discover.nci.nih.gov/cellminer/loadDownload.do) databases. Gene and cell line overlap was performed using the “vlookup” tool in Microsoft Excel. Venn diagrams showing overlap between cancer cell lines and genes in the databases were created using the Venn Diagram Plotter (https://omics.pnl.gov/software/venn-diagram-plotter).
(PDF)

S2 Fig SLFN11 mRNA levels are strongly correlated with sensitivity to chemotherapeutics in cancer cells. A) SLFN11 mRNA levels were compared with drug sensitivity (AUC) in the GDSC dataset by Pearson correlation. Each point represents one drug. Mean ± SEM for each drug class is shown. B-E) Waterfall plots show Pearson’s R for the correlations between SLFN11 mRNA expression and AUC or IC50 for each chemotherapeutic in GDSC, CTRP, and NCI60 datasets. Bars are color-coded according to drug class: green = topoisomerase inhibitor; blue = anti-tumor antibiotic; purple = antimetabolite; red = cytoskeleton inhibitor; orange = alkylating agent. Summarized data are shown in Fig 1B and panel (A) in this supplemental figure.
(PDF)

S3 Fig Pathways analysis of sets of genes predictivity of drug sensitivity and resistance. Genes listed in Table 2, which were correlated with sensitivity (R≤-0.2) or resistance (R≥0.2) to >50% of drugs within a class in ≥2 databases (CTRP, GDSC, or NCI60), were evaluated by unsupervised sample-wise enrichment analysis using the hallmark gene set collection in Gene Set Variation Analysis (GSVA). Adjusted p-values are shown. Hallmark gene sets associated with drug sensitivity (solid shapes) and drug resistance (hollow shapes) are indicated.
(PDF)

S4 Fig SLFN11 mRNA expression in cancer cell lines is significantly correlated between datasets. Gene expression was downloaded from CTRP v2, GDSC, and NCI60 databases as previously described. Each point represents a cell line overlapping between two databases: GDSC vs CTRP (A), CTRP vs NCI60 (B), and GDSC vs NCI60 (C). Pearson’s correlations were performed. Solid line represents the best-fit linear regression line, and dotted lines represent the 95% confidence interval.
(PDF)

S5 Fig SLFN11 is variably expressed across tumor types in cell lines and tumors. A-C) SLFN11 mRNA expression in cell lines separated by cancer cell lineage (solid tumors only) for CTRP (A), GDSC (B), and NCI60 (C). Horizontal lines indicate mean +/- SD. Number of cell lines analyzed for each tissue type are listed in parenthesis. D) SLFN11 mRNA expression in different solid tumor types in TCGA, copied from the Human Protein Atlas (https://www.proteinatlas.org/). Box plots are shown as median and 25th and 75th percentiles. Points are displayed as outliers if they are above or below 1.5 times the interquartile range. Number of tumors analyzed for each tissue type are listed in parenthesis.
(PDF)

S6 Fig SLFN11 mRNA expression is not predictive of overall survival in most cancer subtypes. The OncoLnc tool (http://www.oncolnc.org/) was used to determine whether SLFN11 expression in primary tumors is associated with overall survival in TCGA datasets. Analyses of 21 cancer subtypes was available. p-values were corrected for all genes in the transcriptome. SLFN11 levels were prognostic of shorter OS (FDR-corrected p≤0.05) in 3/21 cancer subtypes.
(PDF)

S1 Table Pearson correlation values for each gene with each drug. Genes with expression that was correlated (R≤0.33) with sensitivity to 4 topoisomerase inhibitor (Camptothecin, Topotecan, Irinotecan, and NSC724998) in Zoppoli et al (2012) are highlighted in red.
(XLSX)
