# Supplementary material for: Integrated pan-cancer gene expression and drug sensitivity analysis reveals SLFN11 mRNA as a solid tumor biomarker predictive of sensitivity to DNA-damaging chemotherapy
Source: PLoS One. 2019 Nov 4;14(11):e0224267. doi: 10.1371/journal.pone.0224267 (PMC6827986; doi:10.1371/journal.pone.0224267)

Supplemental Data

S1 Fig. Genes and solid tumor cell line overlap between cell line databases. RMA-normalized basal expression profiles for cell lines was downloaded from CTRP v2 (https://ocg.cancer.gov/programs/ctd2/data-portal) and GDSC (https://www.cancerrxgene.org/downloads). Z-score-normalized gene expression profiles for cell lines was downloaded from NCI60 (https://discover.nci.nih.gov/cellminer/loadDownload.do) databases. Gene and cell line overlap was performed using the "vlookup" tool in Microsoft Excel. Venn diagrams showing overlap between cancer cell lines and genes in the databases were created using the Venn Diagram Plotter (https://omics.pnl.gov/software/venn-diagram-plotter).

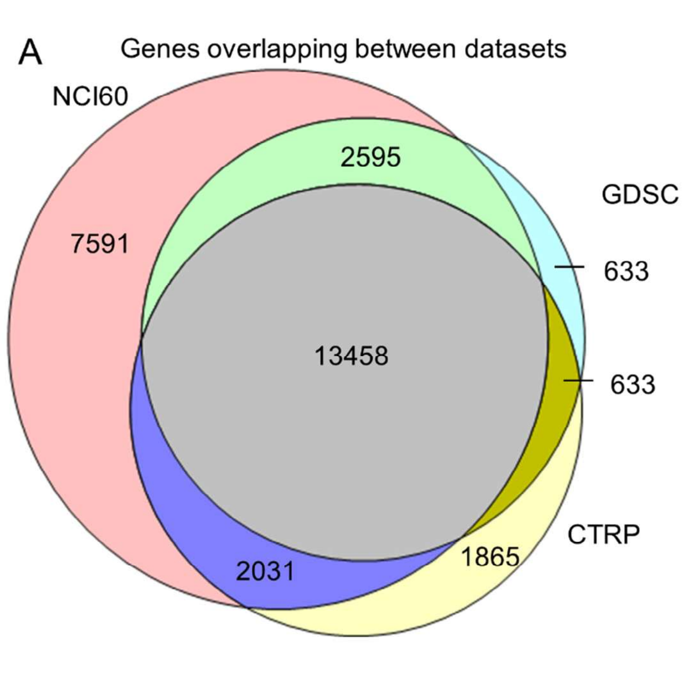

Supplement: S1 Fig — RMA-normalized basal expression profiles for cell lines was downloaded from CTRP v2 (https://ocg.cancer.gov/programs/ctd2/dataportal) and GDSC (https://www.cancerrxgene.org/downloads). Z-score-normalized gene expression profiles for cell lines was downloaded from NCI60 (https://discover.nci.nih.gov/cellminer/loadDownload.do) databases. Gene and cell line overlap was performed using the “vlookup” tool in Microsoft Excel. Venn diagrams showing overlap between cancer cell lines and genes in the databases were created using the Venn Diagram Plotter (https://omics.pnl.gov/software/venn-diagram-plotter). (PDF) [file pone.0224267.s001.pdf]
