# Supplementary material for: Integrated pan-cancer gene expression and drug sensitivity analysis reveals SLFN11 mRNA as a solid tumor biomarker predictive of sensitivity to DNA-damaging chemotherapy
Source: PLoS One. 2019 Nov 4;14(11):e0224267. doi: 10.1371/journal.pone.0224267 (PMC6827986; doi:10.1371/journal.pone.0224267)

S2 Fig. SLFN11 mRNA levels are strongly correlated with sensitivity to chemotherapeutics in cancer cells. A) SLFN11 mRNA levels were compared with drug sensitivity (AUC) in the GDSC dataset by Pearson correlation. Each point represents one drug. Mean ± SEM for each drug class is shown. B-E) Waterfall plots show Pearson's R for the correlations between SLFN11 mRNA expression and AUC or IC_{50} for each chemotherapeutic in GDSC, CTRP, and NCI60 datasets. Bars are color-coded according to drug class: green = topoisomerase inhibitor; blue = anti-tumor antibiotic; purple = antimetabolite; red = cytoskeleton inhibitor; orange = alkylating agent. Summarized data are shown in Fig. 1B and panel (A) in this supplemental figure.

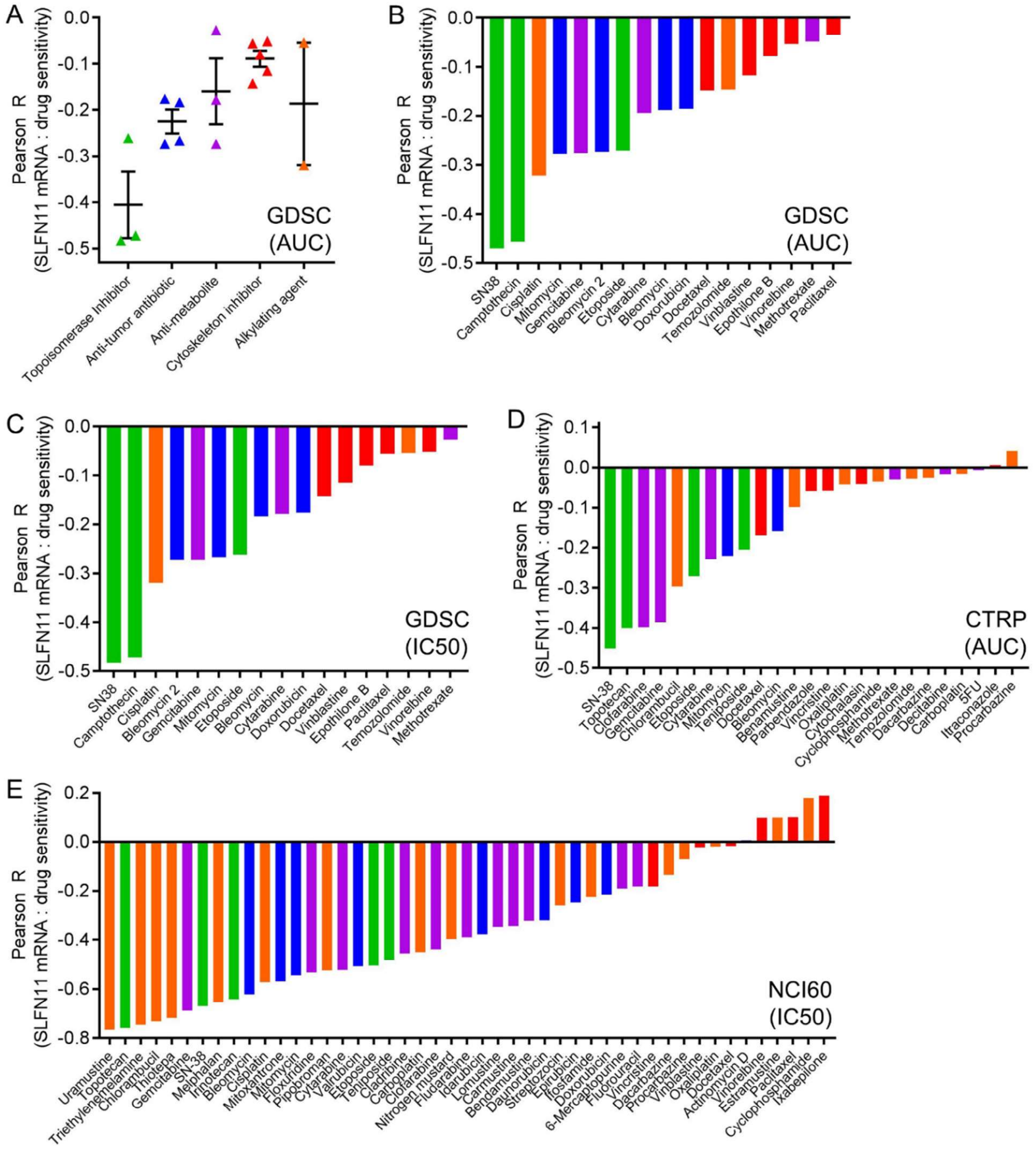

Supplement: S2 Fig — A) SLFN11 mRNA levels were compared with drug sensitivity (AUC) in the GDSC dataset by Pearson correlation. Each point represents one drug. Mean ± SEM for each drug class is shown. B-E) Waterfall plots show Pearson’s R for the correlations between SLFN11 mRNA expression and AUC or IC50 for each chemotherapeutic in GDSC, CTRP, and NCI60 datasets. Bars are color-coded according to drug class: green = topoisomerase inhibitor; blue = anti-tumor antibiotic; purple = antimetabolite; red = cytoskeleton inhibitor; orange = alkylating agent. Summarized data are shown in Fig 1B and panel (A) in this supplemental figure. (PDF) [file pone.0224267.s002.pdf]
