# Supplementary material for: Integrated pan-cancer gene expression and drug sensitivity analysis reveals SLFN11 mRNA as a solid tumor biomarker predictive of sensitivity to DNA-damaging chemotherapy
Source: PLoS One. 2019 Nov 4;14(11):e0224267. doi: 10.1371/journal.pone.0224267 (PMC6827986; doi:10.1371/journal.pone.0224267)

**S3 Fig. Pathways analysis of sets of genes predictivity of drug sensitivity and resistance.** Genes listed in Table 2, which were correlated with sensitivity (R≤-0.2) or resistance (R≥0.2) to >50% of drugs within a class in ≥2 databases (CTRP, GDSC, or NCI60), were evaluated by unsupervised sample-wise enrichment analysis using the hallmark gene set collection in Gene Set Variation Analysis (GSVA). Adjusted *p*-values are shown. Hallmark gene sets associated with drug sensitivity (solid shapes) and drug resistance (hollow shapes) are indicated.

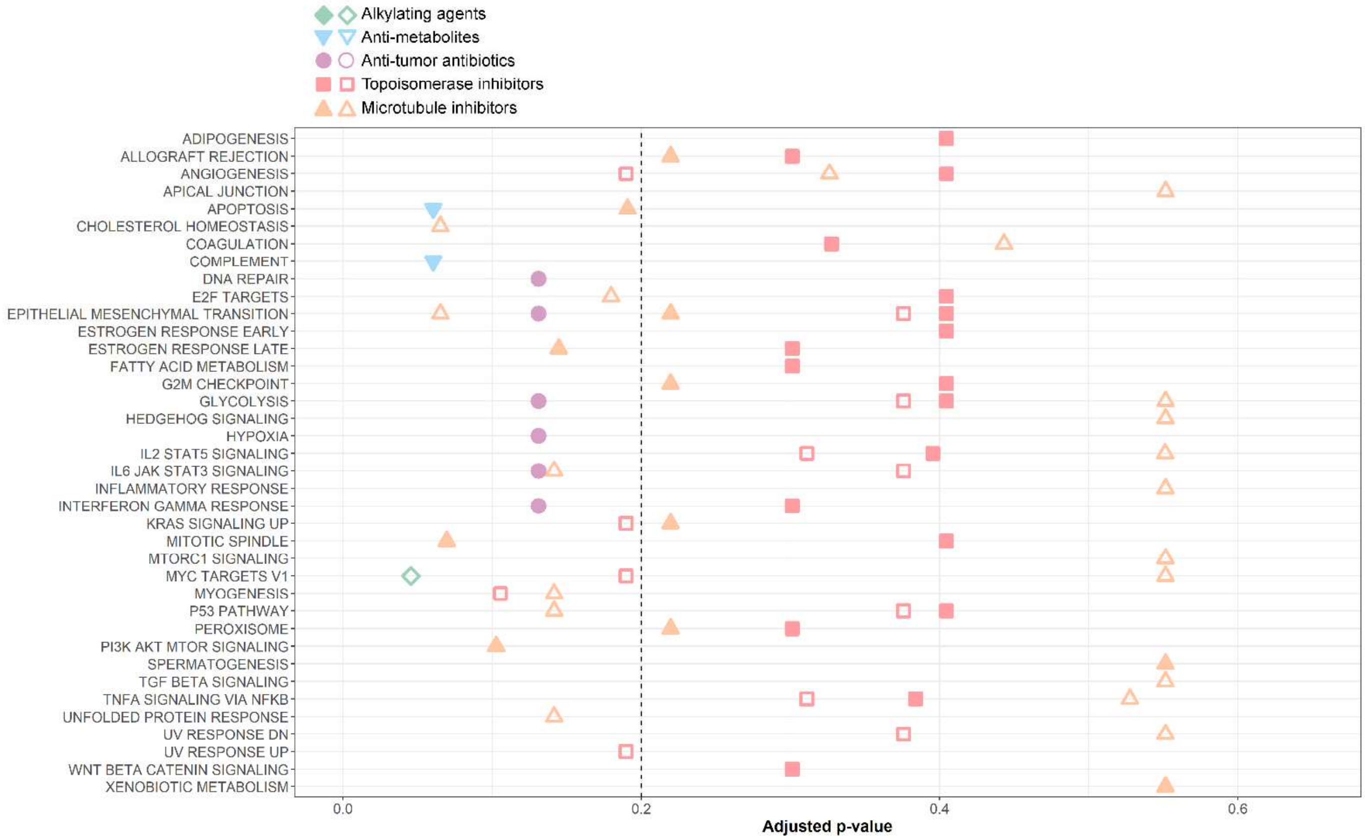

Supplement: S3 Fig — Genes listed in Table 2, which were correlated with sensitivity (R≤-0.2) or resistance (R≥0.2) to >50% of drugs within a class in ≥2 databases (CTRP, GDSC, or NCI60), were evaluated by unsupervised sample-wise enrichment analysis using the hallmark gene set collection in Gene Set Variation Analysis (GSVA). Adjusted p-values are shown. Hallmark gene sets associated with drug sensitivity (solid shapes) and drug resistance (hollow shapes) are indicated. (PDF) [file pone.0224267.s003.pdf]
