# Supplementary material for: Integrated pan-cancer gene expression and drug sensitivity analysis reveals SLFN11 mRNA as a solid tumor biomarker predictive of sensitivity to DNA-damaging chemotherapy
Source: PLoS One. 2019 Nov 4;14(11):e0224267. doi: 10.1371/journal.pone.0224267 (PMC6827986; doi:10.1371/journal.pone.0224267)

S4 Fig. SLFN11 mRNA expression in cancer cell lines is significantly correlated between datasets. Gene expression was downloaded from CTRP v2, GDSC, and NCI60 databases as previously described. Each point represents a cell line overlapping between two databases: GDSC vs CTRP (A), CTRP vs NCI60 (B), and GDSC vs NCI60 (C). Pearson's correlations were performed. Solid line represents the best-fit linear regression line, and dotted lines represent the 95% confidence interval.

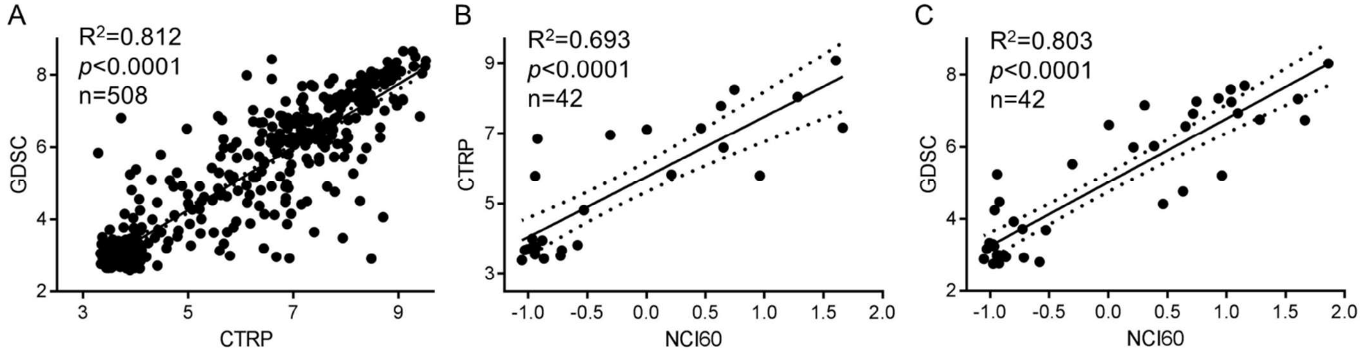

Supplement: S4 Fig — Gene expression was downloaded from CTRP v2, GDSC, and NCI60 databases as previously described. Each point represents a cell line overlapping between two databases: GDSC vs CTRP (A), CTRP vs NCI60 (B), and GDSC vs NCI60 (C). Pearson’s correlations were performed. Solid line represents the best-fit linear regression line, and dotted lines represent the 95% confidence interval. (PDF) [file pone.0224267.s004.pdf]
