# Supplementary material for: Integrated pan-cancer gene expression and drug sensitivity analysis reveals SLFN11 mRNA as a solid tumor biomarker predictive of sensitivity to DNA-damaging chemotherapy
Source: PLoS One. 2019 Nov 4;14(11):e0224267. doi: 10.1371/journal.pone.0224267 (PMC6827986; doi:10.1371/journal.pone.0224267)

S5 Fig. **SLFN11 is variably expressed across tumor types in cell lines and tumors.** A-C) SLFN11 mRNA expression in cell lines separated by cancer cell lineage (solid tumors only) for CTRP (A), GDSC (B), and NCI60 (C). Horizontal lines indicate mean +/- SD. Number of cell lines analyzed for each tissue type are listed in parenthesis. D) SLFN11 mRNA expression in different solid tumor types in TCGA, copied from the Human Protein Atlas (https://www.proteinatlas.org/). Box plots are shown as median and 25th and 75th percentiles. Points are displayed as outliers if they are above or below 1.5 times the interquartile range. Number of tumors analyzed for each tissue type are listed in parenthesis.

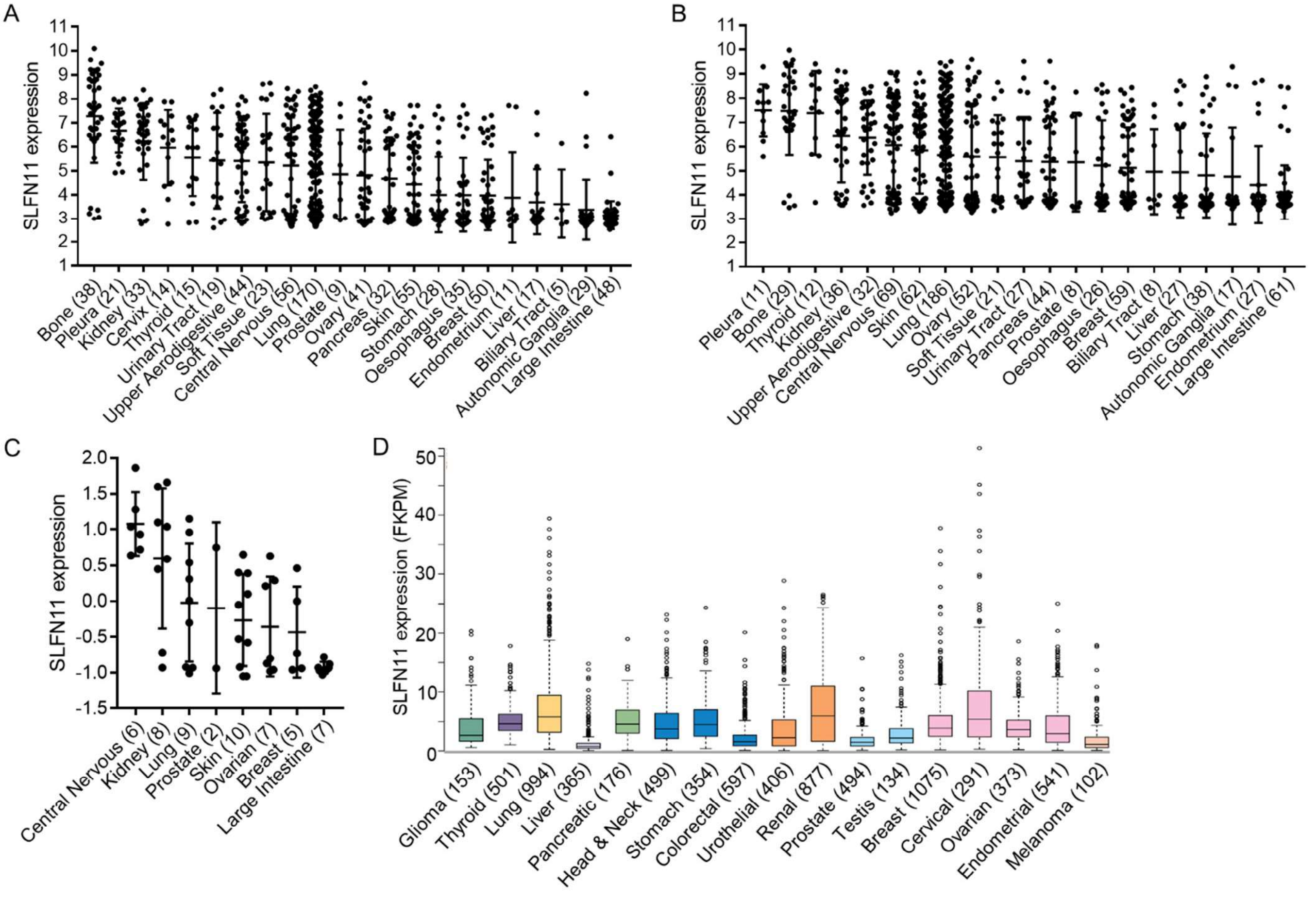

Supplement: S5 Fig — A-C) SLFN11 mRNA expression in cell lines separated by cancer cell lineage (solid tumors only) for CTRP (A), GDSC (B), and NCI60 (C). Horizontal lines indicate mean +/- SD. Number of cell lines analyzed for each tissue type are listed in parenthesis. D) SLFN11 mRNA expression in different solid tumor types in TCGA, copied from the Human Protein Atlas (https://www.proteinatlas.org/). Box plots are shown as median and 25th and 75th percentiles. Points are displayed as outliers if they are above or below 1.5 times the interquartile range. Number of tumors analyzed for each tissue type are listed in parenthesis. (PDF) [file pone.0224267.s005.pdf]
