# Supplementary material for: Integrated pan-cancer gene expression and drug sensitivity analysis reveals SLFN11 mRNA as a solid tumor biomarker predictive of sensitivity to DNA-damaging chemotherapy
Source: PLoS One. 2019 Nov 4;14(11):e0224267. doi: 10.1371/journal.pone.0224267 (PMC6827986; doi:10.1371/journal.pone.0224267)
Supplement: S6 Fig — The OncoLnc tool (http://www.oncolnc.org/) was used to determine whether SLFN11 expression in primary tumors is associated with overall survival in TCGA datasets. Analyses of 21 cancer subtypes was available. p-values were corrected for all genes in the transcriptome. SLFN11 levels were prognostic of shorter OS (FDR-corrected p≤0.05) in 3/21 cancer subtypes. (PDF) [file pone.0224267.s006.pdf]

S6 Fig- *SLFN11* mRNA expression is not predictive of overall survival in most cancer subtypes. The OncoLnc tool [http://www.oncolnc.org/ ; Anaya (2016) OncoLnc: linking TCGA survival data to mRNAs, miRNAs, and lncRNAs. *PeerJ Computer Science* 2: e67] was used to determine whether *SLFN11* expression in primary tumors is associated with overall survival in TCGA datasets. Analyses of 21 cancer subtypes was available. p-values were corrected for all genes in the transcriptome. SLFN11 levels were prognostic of shorter OS (FDR-corrected p≤0.05) in 3/21 cancer subtypes.

Cancer type	TCGA type	FDR-corrected p-value
Bladder urothelial carcinoma	BLCA	0.309
Breast invasive carcinoma	BRCA	0.882
Cervical sq. cell carc. & endocerv. adenocarc.	CESC	0.804
Colon adenocarcinoma	COAD	0.558
Esophageal carcinoma	ESCA	0.988
Glioblastoma multiforme	GBM	0.8
Head & neck squamous cell carcinoma	HNSC	0.594
Kidney renal clear cell carcinoma	KIRC	0.022
Kidney renal papillary cell carcinoma	KIRP	0.123
Acute myeloid leukemia	LAML	0.736
Brain lower grade glioma	LGG	0.023
Liver hepatocellular carcinoma	LIHC	0.654
Lung adenocarcinoma	LUAD	0.944
Lung squamous cell carcinoma	LUSC	0.567
Ovarian serous cystadenocarcinoma	OV	0.978
Pancreatic adenocarcinoma	PAAD	0.938
Rectum adenocarcinoma	READ	0.932
Sarcoma	SARC	0.505
Skin cutaneous melanoma	SKCM	0.002
Stomach adenocarcinoma	STAD	0.641
Uterine corpus endometrial carcinoma	UCEC	0.883
